# The Prevalence of Difficult Vascular Anatomy in Transulnar Versus Transradial Access for Cardiac Catheterization in Propensity Score‐Matched Cohorts

**DOI:** 10.1002/hsr2.71037

**Published:** 2025-07-10

**Authors:** Mona Maria Grewe, Franziska Fochler, Peter Hubert Grewe, Lars Maier, Christian Schmidt, Kirstin Meier, Tobias Roeschl

**Affiliations:** ^1^ Clinic of Cardiology and Angiology, Klinikum Neumarkt Neumarkt in der Oberpfalz Germany; ^2^ Department of Internal Medicine II University Hospital Regensburg Regensburg Germany

**Keywords:** difficult vascular anatomy, radial artery atherosclerosis, transulnar access, ulnar artery, ultrasound guided access

## Abstract

**Background and Aims:**

Transradial access (TRA) is used with a Class IA recommendation for coronary angiography (CA) or percutaneous coronary intervention (PCI). Difficult vascular anatomy (DVA) of the forearm arteries is a challenge to its success. Transulnar artery access (TUA) may constitute a viable alternative.

**Methods:**

In this single‐center study, we retrospectively compared the prevalence of DVA at the forearm arteries in 2565 consecutive cases of CA/PCI (2403 TRA and 162 TUA) between 2019 and 2022. DVA was classified as zeroth‐degree if the forearm could be passed with a standard 0.035″ guidewire. First‐degree was defined if a standard 0.035″ guidewire had to be switched to a hydrophilic 0.035″ or a 0.018″ guidewire to successfully reach the brachial artery and second‐degree DVA if the forearm arteries could not be passed with any guidewire leading to procedural failure.

**Results:**

In the overall cohort, DVA was significantly more prevalent in TRA versus TUA (zeroth‐degree, first‐degree, second‐degree: 90.3%, 7.2%, and 2.5% vs. 96.9%, 3.1%, and 0%, respectively, *p* = 0.008). After 4:1 propensity score matching, second‐degree DVA remained more prevalent in TRA versus TUA (2.9% (*n* = 648) vs. 0% (*n* = 162, *p* < 0.001)).

**Discussion:**

In our retrospective analysis, primary TUA was found to be superior to TRA regarding DVA at the forearm arteries. TUA may present a superior alternative to TRA for CA/PCI in the era of ultrasound‐guided arterial access.

**Abbreviations**
CA: coronary angiography
DVA: difficult vascular anatomy
PCI: percutaneous coronary intervention
RA: radial artery
TFA: transfemoral access
TRA: transradial access
TUA: transulnar access
UA: ulnar artery

## 1 Introduction

Access routes for coronary angiography (CA) or percutaneous coronary interventions (PCI) have shown continuous progress in recent years. In particular, transradial access (TRA) has gained importance [1], showing a reduction in puncture site‐related bleeding complications and all‐cause mortality compared to transfemoral access (TFA) [2]. Therefore, TRA is recommended as the primary arterial access for CA/PCI according to the latest guidelines (“radial‐first approach”) [1, 3, 4].

**Figure 1:**
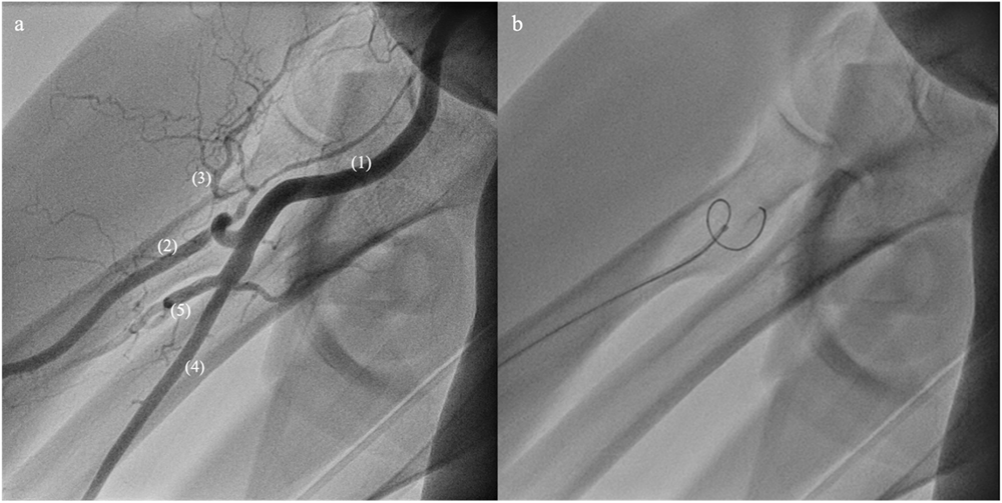
(a) Single shot picture from a standardized forearm angiography, displayed arteries: (1) brachial artery, (2) radial artery with 360° loop, (3) radial collateral artery, (4) ulnar artery, (5) interosseous artery. (b) 0.018″ guidewire in 360° radial loop, termination of procedure due to second‐degree DVA, as no further wire advancement was possible.

However, TRA also has limitations as it is complicated by atherosclerosis of the radial artery (RA) in the forearm and vascular variants in the radio‐ulnar‐brachial region. These variations include tortuosity, loops, and a high origin of the RA potentially impeding guidewire advancement to the brachial artery or leading to unintended cannulation of the radial collateral artery [5, 6]. Misintubation of the radial collateral artery occurs particularly in cases where the RA forms a loop immediately after branching off from the brachial artery (Figure 1a) [7]. In addition, complicated guidewire passage is associated with vascular complications such as arterial dissection, which can lead to acute arm ischemia, bleeding, or compartment syndrome [8].

The prevalence of vascular variants complicating CA/PCI in the radio‐ulnar‐brachial region has been reported to be 2%–12% in previous studies. Therefore, alternative access routes were investigated [5, 9, 10, 11].

The first successful CA via the ulnar artery (UA) was performed in 2001 [12]. Subsequent studies have demonstrated that transulnar access (TUA) is safe and can be used in patients in whom the RA is occluded, has been or will be used for coronary artery bypass grafting or in arteriovenous fistula procedures [13, 14, 15].

Due to the UA's deep path within the muscles of the anterior forearm, it is less palpable and thus more challenging to cannulate compared to the RA. However, the widespread use of ultrasound‐guided access alleviates this anatomical challenge [16]. In addition, there is growing evidence in the literature that some vascular variants, such as loops and tortuosity, are predominantly found in the RA but not in the UA [17, 18]. Thus, TUA may be associated with higher procedural success rates compared to TRA once arterial access is established.

In our study, we compared the prevalence of vascular variants complicating guidewire passage in the RA and UA after ultrasound‐guided TRA versus TUA before and after propensity score matching. Specifically, we assessed (a) the prevalence of first‐ or second‐degree DVA (“DVA present”) and (b) the prevalence of second‐degree DVA (“DVA leading to procedural failure”) in the forearm arteries between TUA and TRA cases.

The secondary outcome of this study included the assessment of intraprocedural complications, such as vasospasm, ischemic or neurological complications, and bleeding events, to evaluate the safety of TRA and TUA.

## 2 Methods

**Figure 2:**
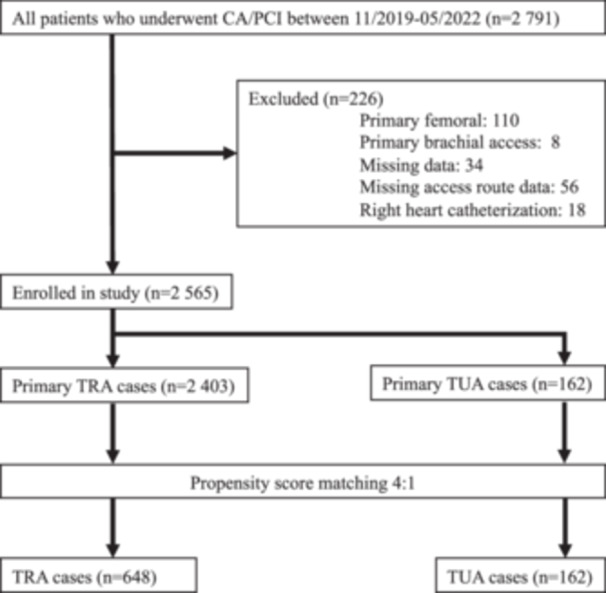
Flowchart illustrating the selection process of cases included in the study. Of 2791 CA/PCI cases performed between November 2019 and May 2022, 226 cases were excluded due to predefined criteria (primary femoral/brachial access, missing data, or right heart catheterization). The final analysis included 2565 cases, categorized into 2403 transradial access and 162 transulnar access.

This retrospective single‐center study analyzed 2791 cases of CA/PCI performed between November 2019 and May 2022. After applying predefined exclusion criteria, 2565 cases were included in the final analysis. All CA/PCI procedures performed during the study period were included. Cases were excluded if they met any of the following criteria: primary femoral access, primary brachial access, missing data (e.g., incomplete procedural documentation), missing access route data, and/or right heart catheterization. Of the 2565 included cases, 2403 were performed via primary TRA, while 162 were performed via primary TUA. A detailed overview of patient selection and exclusion is provided in Figure 2.

The study was conducted at *Klinikum Neumarkt*, an academic teaching hospital affiliated with Friedrich‐Alexander‐University Erlangen‐Nürnberg. Accordingly, ethical approval was obtained from the Ethics Committee of Friedrich‐Alexander‐University Erlangen‐Nürnberg, which is responsible for overseeing research at its affiliated institutions (20‐548_1‐Br). The approval included the collection of data on implied consent owing to the retrospective and observational nature of the study.

### 2.1 Technical Considerations

Homogeneity in procedural aspects, techniques and materials used, were maintained over the study period. The operator team consisted of six physicians (three seniors, two juniors, and one trainee) throughout the duration of data acquisition. Seniors were classified as having performed > 1000 CAs/PCIs via forearm artery access prior to the study, juniors as having performed 250–1000 procedures and trainees as having performed < 250 procedures. All procedures were executed under the supervision of a senior physician. The choice of access site and forearm artery was determined by the operator based on preprocedural ultrasound assessment, considering vessel diameter and the presence of plaques or calcification at the intended puncture site. Arterial access sites included the proximal RA, the distal RA in the anatomical snuffbox, and the UA.

TRA was performed as described previously [5, 19]. Briefly, after positioning the forearm on a carbon fiber board (Starboard, Adept Medical Ltd., Auckland, New Zealand), the RA was punctured using a 0.8 × 50 mm G21 needle (B. Braun SE, Melsungen, Germany). The ultrasound probe (9L probe, Vivid T9, GE Healthcare, Boston, Massachusetts) was enveloped in a sterile cover (Flexasoft, Udo Heisig GmbH, Putzbrunn, Germany) and RA puncture was performed under out‐of‐plane ultrasound guidance. A 0.021‐inch, 45‐cm wire was advanced into the arterial lumen, followed by the placement of a 70‐mm hydrophilic‐coated introducer sheath (6 French Glidesheath Slender, Terumo Europe N.V., Leuven, Belgium). After sheath insertion, 2 mg of verapamil and 5000 IU of heparin were administered through the sheath. Subsequently, angiography of the distal upper arm and the forearm, extending to the wrist, was performed. With knowledge of the anatomy of the forearm arteries, a 0.035″ wire (Angiodyn, B. Braun SE) was advanced to the upper arm under continuous fluoroscopic guidance.

### 2.2 Modified Transulnar Access

On the wrist the UA shows a physiological kinking, which straightens after dorsiflexion of the hand. In its proximal course, the UA first follows the tendon of the flexor carpi ulnaris and then arches underneath its muscle belly into the depth. In this section the UA often shows calcifying atherosclerosis.

In our modified approach for TUA, those two anatomical obstacles are passed with an atraumatic hydrophilic 0.014″ PCI wire (Choice PT floppy, Boston Scientific, Marlborough, USA). Prior to this study, we observed that the 0.014″ wire is more successful in passing through atherosclerotically altered vessel segments than the standard sheath wire (Glidesheath Slender, Terumo Europe N.V., Leuven, Belgium).

TUA was obtained according to the above‐described TRA: A 0.8 × 50 mm G21 needle was used for puncture, and in contrast to TRA, the 0.014″ wire was advanced into the lumen of the UA. Subsequently, a 70‐mm hydrophilic coated introducer sheath was introduced. Upon successful sheath insertion, 2 mg of verapamil and 5000 IU of heparin were administered via the sheath. If the duration exceeded 20 min, an activated clotting time adjusted heparin therapy was initiated (> 200 s). With every procedure, a standardized forearm angiography, visualizing the mid‐upper arm and the forearm up to the wrist, was performed as described previously (Figure 1) [19]. Under continuous fluoroscopic guidance a 0.035″ J wire was advanced to the upper arm via the sheath in analogy with the standard TRA as described previously [5]. The puncture site was closed with a RA compression device (TR Band, Terumo Europe N.V.).

### 2.3 Defining Difficult Vascular Anatomy

The classification of DVA used in our study provides a purely functional assessment, i.e., the ability to pass the forearm arteries with a guidewire and does not include visual aspects (e.g., the severity of vessel tortuosity) obtained during forearm angiography [5].

DVA of the forearm arteries (from the puncture site to the radio‐ulnar‐brachial region) was divided into three categories [5]. In short, no DVA (zeroth‐degree DVA) was defined if the forearm could be passed with a standard 0.035″ guidewire. First‐degree DVA was characterized by vessels exhibiting tortuosity, stenosis, or calcification requiring the use of a hydrophilic 0.035″ guidewire (Radifocus Guidewire M, Terumo Europe N.V.) and/or a 0.018″ guidewire (Advantage, Terumo Europe N.V., Figure 1b). Second‐degree DVA encompassed severely tortuous, calcified, or occluded vessels that proved to be impassable with any guidewire, resulting in procedural failure and the need to switch to an alternative access site. After each procedure, the operating physician documented the severity of DVA at the forearm.

### 2.4 Statistical Analysis

For statistical analysis, Q–Q plots and the Shapiro–Wilk test were used to assess normality. Continuous variables were summarized as mean ± standard deviation (SD) and categorical variables as percentages (count). Student's *t*‐test was used for continuous data and *χ* ^2^ test for categorical data.

Due to imbalances in potential confounding variables between primary TUA and primary TRA cases, we used *k*:1‐nearest neighbor propensity score matching. Propensity scores were calculated by logistic regression with predefined variables (age, height, body mass index, sex, operator experience, clinical urgency, arterial hypertension, diabetes mellitus, left vs. right forearm artery access, and smoking status). The optimum matching ratio (*k*) was determined so that the maximum standardized mean difference of any confounder was minimized. This resulted in a matching ratio of 4:1. We applied the nearest‐neighbor algorithm without replacement and used a caliper width of 0.25. The balance of covariates was considered satisfactory for a standardized mean difference of < 0.1.

After propensity score matching, we compared the prevalences of both outcomes through linear risk difference estimation and calculated standard errors using a cluster‐robust technique [20]. To meet the assumption of independence, only the first access attempt was included to determine the prevalence of DVA, that is, if right TRA failed due to, for example, a radial loop and crossover to right TUA was necessary, observations from right TUA were not included in the analysis. All statistical analyses were carried out with Python 3.9 and R 4.0.3.

## 3 Results

In this retrospective analysis, we compared the success rate of TRA and TUA for CA/PCI with respect to DVA of the RA and UA. In total, 2565 cases, encompassing 2403 TRA and 162 TUA procedures for primary arterial access, were analyzed (Figure 2).

### 3.1 Unmatched Cohorts

The study population had a mean age of 69.6 ± 12.0 years and 824 (32.1%) patients were female. In the unmatched cohort, the TUA group did not differ significantly from the TRA group in terms of patient age (70.4 ± 10.5 vs. 69.5 ± 12.1 years, *p* = 0.381), height (171.7 ± 9.0 vs. 171.6 ± 9.3 cm, *p* = 0.864), body mass index (28.9 ± 5.8 vs. 28.3 ± 5.3 kg/m^2^, *p* = 0.168), female gender (32.1 vs. 32.1%, *p* = 1.0), smoking history (26.5% vs. 28.0%, *p* = 0.764), and diabetes mellitus (23.5 vs. 22.1%, *p* = 0.770). The TUA group differed significantly from the TRA group in terms of arterial hypertension (100.0 vs. 92.3%, *p* < 0.001), left FAA (33.3 vs. 53.3%, *p* < 0.001), operator experience, clinical urgency and the year of intervention (Table 1). Zeroth‐degree DVA was observed in 2171 (90.3%) TRA and 157 (96.9%) TUA cases, first‐degree DVA in 172 (7.2%) TRA and 5 (3.1%) TUA cases, and second‐degree DVA in 60 (2.5%) TRA and 0 (0%) TUA cases (*p* = 0.015, Table 2). Overall, in 232 (9.7%) TRA versus 5 (3.1%) TUA cases either first‐ or second‐degree DVA was observed (*p* = 0.008, Table 2).

**Table 1:** Pre‐ and post‐matching results for continuous values mean ± standard deviation (SD) and *n* (%) for dichotomous data.

	Pre‐matching			Post‐matching	
	TRA (*n* = 2403)	TUA (*n* = 162)	*p*	TRA (*n* = 648)	TUA (*n* = 162)
Age, years	69.5 ± 12.1	70.4 ± 10.5	0.381	70.4 ± 11.6	70.4 ± 10.5
Height, cm	171.6 ± 9.3	171.7 ± 9.0	0.864	171.9 ± 9.6	171.7 ± 9.0
Female gender	772 (32.1)	52 (32.1)	1.000	201 (31.3)	52 (32.1)
Weight, kg	83.5 ± 18.0	85.3 ± 18.6	0.229	84.5 ± 19.1	85.3 ± 18.5
BMI, kg/m^2^	28.3 ± 5.3	28.9 ± 5.8	0.168	28.8 ± 5.9	28.9 ± 5.7
Smoking history	672 (28.0)	43 (26.5)	0.764	166 (25.6)	43 (26.5)
Diabetes mellitus	532 (22.1)	38 (23.5)	0.770	172 (26.5)	38 (23.5)
Arterial hypertension	2 217 (92.3)	162 (100.0)	**< 0.001**	648 (100.0)	162 (100.0)
Left FAA, *n* (%)	1 368 (53.3)	54 (33.3)	**< 0.001**	205 (31.6)	54 (33.3)
Operator experience					
Senior	1 590 (66.2)	146 (90.1)	**< 0.001**	580 (89.5)	146 (90.1)
Junior	710 (29.5)	10 (6.2)		38 (5.9)	10 (6.2)
Trainee	103 (4.3)	6 (3.7)		30 (4.6)	6 (3.7)
Clinical urgency					
Elective	1 669 (69.5)	139 (85.8)	**0.002**	547 (84.4)	139 (85.8)
Cardiac arrest	4 (0.2)	0 (0.0)		—	—
Cardiogenic shock	57 (2.4)	1 (0.6)		9 (1.4)	1 (0.6)
Unstable angina	75 (3.1)	3 (1.9)		11 (1.7)	3 (1.9)
NSTEMI	395 (16.6)	12 (7.5)		55 (8.6)	12 (7.5)
STEMI	203 (8.5)	7 (4.4)		21 (3.3)	7 (4.4)
Year of intervention					
2019	171 (7.1)	20 (12.3)	**< 0.001**	53 (8.2)	20 (12.3)
2020	853 (35.5)	34 (21.0)		238 (36.7)	34 (21.0)
2021	878 (36.5)	58 (35.8)		226 (34.9)	58 (35.8)
2022	501 (20.8)	50 (30.9)		131 (20.2)	50 (30.9)

Bold values indicate statistically significant *p* < 0.05.

Abbreviations: ACS, acute coronary syndrome; BMI, body mass index; NSTEMI, non‐ST‐elevation myocardial infarction; STEMI, ST‐elevation myocardial infarction; TRA, transradial access; TUA, transulnar access.

**Table 2:** Pre‐matching results for DVA at the forearm arteries.

	Pre‐matching		
	TRA (*n* = 2403)	TUA (*n* = 162)	*p*
DVA at the forearm arteries			
Zeroth‐degree	2 171 (90.3)	157 (96.9)	**0.015**
First‐degree	172 (7.2)	5 (3.1)	
Second‐degree	60 (2.5)	0 (0.0)	
First‐ or second‐degree	232 (9.7)	5 (3.1)	**0.008**

*Note:* Variables are expressed as *n* (%). Bold values indicate statistically significant *p* < 0.05.

Abbreviations: DVA, difficult vascular anatomy; TRA, transradial access; TUA, transulnar access.

### 3.2 Matched Cohorts

**Figure 3:**
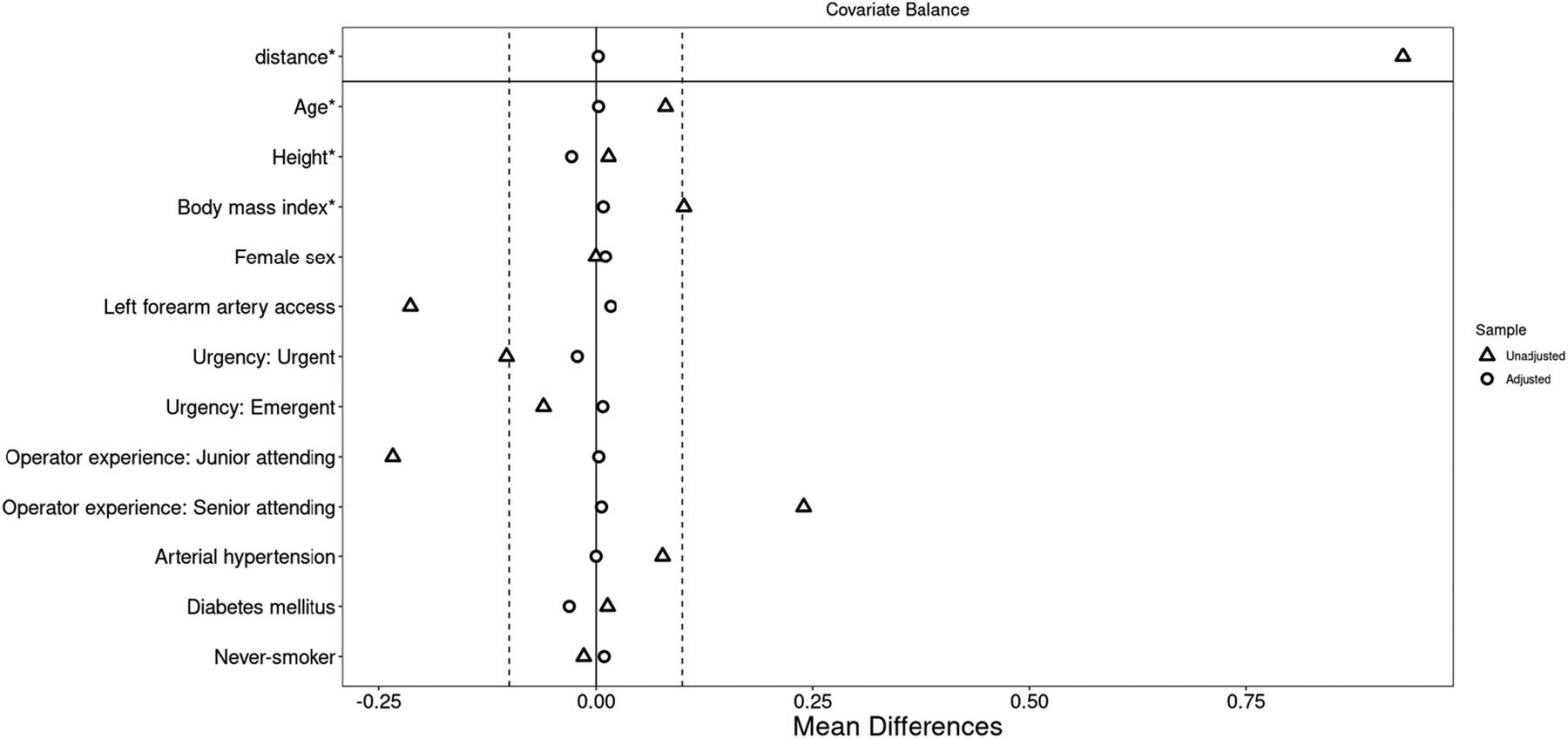
Love plot figure showing the covariate balance between unmatched (triangle) and matched (circle) covariates. The horizontal axis represents mean differences between treatment groups. For covariates marked with an asterisk (*), differences are expressed as standardized mean differences (SMDs); for others, raw mean differences are shown.

After propensity score matching, the two groups were well balanced regarding all variables (Figure 3). In the matched groups, second‐degree DVA was observed in 19 (2.9%) TRA cases and 0 (0%) TUA cases (*p* < 0.001). As second‐degree DVA corresponds to procedural failure, we observed a crossover rate of 2.9% in the TRA group and 0% in the TUA group (*p* < 0.001). The combined outcome of either first‐ or second‐degree DVA was observed in 52 (8.0%) TRA and 5 (3.1%) TUA cases (*p* = 0.005, Table 3).

**Table 3:** Post‐matching results for DVA at the forearm arteries.

	Post‐matching		
DVA at the forearm arteries	TRA (*n* = 648)	TUA (*n* = 162)	*p*
Second‐degree	19 (2.9)	0 (0.0)	**< 0.001**
First‐ or second‐degree	52 (8.0)	5 (3.1)	**0.005**

*Note:* Variables are expressed as *n* (%). Bold values indicate statistically significant *p* < 0.05.

Abbreviations: DVA, difficult vascular anatomy; TRA, transradial access; TUA, transulnar access.

### 3.3 Vascular Complications

We observed no ischemic or neurologic complications and no case of vasospasm during 162 TUA cases. In 17 (10.5%) TUA cases a local hematoma could be observed, which was treated conservatively.

## 4 Discussion

This study compared the prevalence of DVA in TRA versus TUA for CA/PCI. Our functional assessment indicates that vascular variants impeding guidewire passage at the forearm arteries were significantly less frequent in TUA compared to TRA. Importantly, second‐degree DVA resulting in access failure was absent in all TUA cases.

### 4.1 Anatomical Differences in the Forearm Arteries Determine Procedural Success Rates

In both, unmatched and matched cohorts, the prevalence of DVA (first‐ and second‐degree) was significantly lower in the TUA group compared to TRA. In the unmatched cohort, second‐degree DVA—a common cause of access failure—was absent in TUA cases, whereas it was present in 2.5% of TRA cases. After matching, second‐degree DVA persisted in the TRA group (2.9%), further demonstrating TUA's anatomical advantage. The lower DVA rate observed in TUA may be explained by anatomical differences between the RA and UA. Lo and colleagues describe the respective prevalences of vascular variants complicating TRA in their study. In this study, the authors report that failure of TRA was highest in cases of radial loops and radial tortuosity followed by atherosclerosis and high origin of the RA [9].

Emerging evidence in the literature suggests these vascular variants are less frequently found in the UA. A potential explanation for this could be that the UA represents the radiographic anatomical continuation of the brachial artery, with its—compared to the RA—larger diameter, straighter course and relatively flat branching angle from the brachial artery potentially making the UA less susceptible to these vascular variants [18, 21, 22]. These anatomical differences between the RA and UA may lead to higher procedural success rates highlighting TUA as a promising alternative.

### 4.2 Beyond the Guidelines There Is a Need for Alternative Access Routes

Current guidelines recommend TRA as a Class IA indication for routine CA/PCI, including cases of acute coronary syndrome [1, 3]. However, our findings underscore the advantages of TUA over TRA, especially when the efficacy of TRA is limited by DVA, like atherosclerosis, tortuosity, loops or a high origin of the RA. Additionally, TRA success rates may be compromised by difficulties in RA localization or a small RA diameter at the puncture site. Previously, TRA may have led to RA occlusion [23] or the RA may have been used or will be needed as a coronary artery bypass grafting or in arteriovenous fistula procedures [24]. In most cases, clinical judgment advocates for TFA; however, TFA shows higher bleeding and mortality rates [23, 25].

The lower prevalence of DVA in the UA suggests that TUA may be a viable alternative access route in patients with anatomical variations of the RA or in those with contraindications for TRA.

### 4.3 Ultrasound Guidance for TUA

In contrast to our results, Hahalis and colleagues described inferiority of TUA compared to TRA due to a higher puncture‐related crossover rate. In this study, however, puncture of the UA was attempted even in cases without sufficient arterial palpability [26].

Another study showed results consistent with our findings, that is, no significant differences in procedural attempts between TUA and TRA. The high success rates in this study were primarily contributed to operator experience. However, TUA was only performed when the UA was easily palpable, which represents a selection bias [27, 28]. The more difficult palpability of the UA due to its anatomical course, represents a disadvantage for the UA as access site. This can be circumvented by the consistent use of ultrasound as was the case in our study. As puncture occurs in the three‐dimensional space, ultrasound provides a real‐time visualization of the target vessel and the needle's trajectory relative to the artery. Therefore, ultrasound guidance is the first key factor in enhancing the procedural reliability of TUA [27, 28, 29, 30]. Additionally, damage to the ulnar nerve, which is located lateral to the UA, can be effectively circumvented [31].

### 4.4 Using a Hydrophilic 0.014″ PCI Wire to Straigthen the Ulnar Artery

The UA exhibits a physiological S‐curve at the level of the wrist and continues with an arched course, running deeper beneath the forearm musculature. These anatomical peculiarities can potentially lead to the inability to advance the standard sheath guidewire or cause arterial perforation during sheath insertion.

In our modified approach for TUA, those two anatomical obstacles are passed with an atraumatic hydrophilic 0.014″ PCI wire which straightens the UA at the forearm and facilitates the advancement of the standard guidewire. Implementation of the hydrophilic 0.014″ wire is the second key factor in our modified TUA.

### 4.5 Standardized Forearm Angiography

In a previous study, we could show that a implementation of a standardized forearm angiography before guidewire advancement increases procedural success [19]. Despite the UA being less affected by DVA, it is reasonable to also perform a forearm angiography during TUA to allow for proactive guidewire adjustment, including an early switch to a hydrophilic guidewire in the presence of DVA.

### 4.6 Procedural Safety

Our secondary outcome analysis focused on intraprocedural complications associated with TUA. In our cohort, we observed no vasospasms, ischemic or neurologic complications—despite the UA's proximity to the ulnar nerve. Local hematoma formation occurred in 10.5% of cases and was treated conservatively, which aligns with previous studies on TUA, where the UA's deeper anatomical position compared to the RA makes it more difficult to achieve hemostasis [14]. With the first key factor of our modified TUA—ultrasound‐guided puncture—the risk of local hematoma can be minimized which is consistent with the literature [14, 26, 32, 33].

Taken together, we demonstrate that TUA has a higher success rate due to a significantly lower DVA rate. Our modified TUA approach demonstrated high procedural success, attributed to ultrasound guidance, application of the 0.014″ wire to navigate the physiological S‐shaped course of the UA at the wrist, and to accommodate the UA's naturally arcuate course along the forearm. Standardized forearm angiography enables proactive guidewire adjustment to the observed vascular variants and avoids misintubation of side branches. Furthermore, our modified TUA was associated with a favorable safety profile, as no major vascular complications were observed. Therefore, this study demonstrates the feasibility of TUA as an alternative to TRA for CA/PCI.

## 5 Limitations

Our study is subject to certain limitations. These include a single‐center patient population, a well‐experienced team of interventional cardiologists performing the procedures and the retrospective nature of our investigation. Randomized controlled trials are needed to fully validate TUA as a standard alternative to TRA. Additionally, long‐term follow‐up is required to assess chronic complications such as UA occlusion and hand ischemia, particularly in high‐risk patients.

## 6 Conclusion

Our study demonstrated that TUA is associated with less DVA at the forearm compared to TRA thus making TUA a viable alternative for primary arterial access for cardiac catheterization. Ultrasound‐guided arterial canulation, using a 0.014″ wire to straighten the UA and consistent use of a standardized forearm angiography have the potential to maintain high procedural success rates in TUA.

## Author Contributions

**Mona Maria Grewe:** conceptualization, data curation, formal analysis, writing – original draft. **Franziska Fochler:** conceptualization, writing – original draft, writing – review and editing. **Peter Hubert Grewe:** conceptualization, methodology, supervision. **Lars Maier:** writing – review and editing. **Christian Schmidt:** writing – review and editing. **Kirstin Meier:** writing – review and editing. **Tobias Roeschl:** formal analysis, writing – review and editing.

## Conflicts of Interest

The authors declare no conflicts of interest.

## Transparency Statement

The lead author Peter Hubert Grewe affirms that this manuscript is an honest, accurate, and transparent account of the study being reported; that no important aspects of the study have been omitted; and that any discrepancies from the study as planned (and, if relevant, registered) have been explained.

## Data Availability

The data that support the findings of this study are available from the corresponding author upon reasonable request.
